# Sir Eramus Wilson’s Munificent Gift to Margate

**Published:** 1882-11

**Authors:** 


### Sir Erasmus Wilson's Munificent Gift to Margate.

At a recent meeting of the Governors of the Margate Royal Sea Bathing Infirmary, Sir Erasmus Wilson, F. R. S., handed over the key of the magnificent new wing of the Infirmary, to be named the Erasmus Wilson wing, which he has built at an estimated cost of £150,000. The wing includes two large day-rooms and four dormitories, each to contain sixteen beds, with a swimming bath capable of containing 15,000 gallons of sea water.

